# Incremental RBF-based cross-tier interference mitigation for resource-constrained dense IoT networks in 5G communication system

**DOI:** 10.1016/j.heliyon.2024.e32849

**Published:** 2024-06-12

**Authors:** Omar Alruwaili, Jaganathan Logeshwaran, Yuvaraj Natarajan, Majed Abdullah Alrowaily, Shobhit K. Patel, Ammar Armghan

**Affiliations:** a Department of Computer Engineering and Networks, College of Computer and Information Sciences, Jouf University, Sakaka, 72388, Saudi Arabia; b Department of Electronics and Communication Engineering, Sri Eshwar College of Engineering, Coimbatore, 641202, Tamil Nadu, India; c Department of Computer Science and Engineering, Sri Shakthi Institute of Engineering and Technology, Coimbatore, Tamil Nadu, 641062, India; d Department of Computer Science, College of Computer and Information Sciences, Jouf University, Sakaka, 72388, Saudi Arabia; e Department of Computer Engineering, Marwadi University, Rajkot, Gujarat, 360003, India; f Department of Electrical Engineering. College of Engineering, Jouf University, Sakaka, 72388, Saudi Arabia

**Keywords:** Cross-tier interference, RBF, IoT, 5G network, Packet loss, Latency

## Abstract

The deployment of resource-constrained and densely distributed Internet of Things (IoT) devices poses significant challenges for 5G communication systems due to the increased likelihood of inter-tier interference. This interference can degrade network performance and hinder the transmission of data in a reliable and efficient manner. Using an incremental Radial Basis Function (RBF) technique, this paper proposes a novel approach for cross-tier interference mitigation in 5G communication among resource-constrained dense IoT networks. Utilizing the incremental RBF method to model and optimize interference patterns in resource-constrained dense IoT networks is the primary innovation of our approach. In contrast to conventional interference mitigation techniques, which view interference as a static phenomenon, our method adapts to the dynamic nature of IoT networks by incrementally updating the RBF model. This enables precise modeling of the various interference scenarios and real-time modification of interference mitigation parameters. Utilizing the spatial distribution of IoT devices, this approach improves interference mitigation. The proposed method intelligently allocates resources and optimizes interference mitigation parameters based on the location and density of IoT devices. This adaptive resource allocation improves network capacity, reliability, and overall system performance by maximizing the utilization of available resources while minimizing interference. We demonstrate the effectiveness of the incremental RBF-based approach in mitigating cross-tier interference in resource-constrained dense IoT networks within the 5G ecosystem through extensive experiments and simulations. Our findings indicate substantial improvements in communication performance, including increased throughput, decreased packet loss, and decreased latency.

## 1 Introduction

Significant growth has occurred in the deployment of IoT devices in 5G communication systems, enabling a variety of applications and services across multiple domains required for smart cities [1]. Nonetheless, the proliferation of resource-constrained and densely distributed IoT devices presents new obstacles to achieving reliable and efficient communication. Cross-tier interference, which occurs when IoT devices interfere with each other and other network components, resulting in degraded performance and compromised data transmission, is one of the most significant obstacles [2]. This interference becomes even more pronounced in dense IoT networks with limited resources, where the lack of resources exacerbates the problem [3]. This research focuses on developing an innovative approach based on incremental Radial Basis Function (RBF) for cross-tier interference mitigation in 5G communication systems among resource-constrained dense Internet of Things (IoT) networks [4]. The unique characteristics of resource-constrained dense IoT networks present specific research gaps that must be addressed despite the growing emphasis on interference mitigation in 5G communications [5]. The dynamic nature of IoT networks, coupled with the limited resources of IoT devices, necessitates the development of novel cross-tier interference management techniques [6]. Existing approaches frequently rely on static interference models or resource allocation strategies that do not adequately account for the spatial distribution and dynamics of IoT devices [7]. Therefore, there is an urgent need to develop a solution capable of accurately modeling and optimizing interference patterns, while efficiently allocating resources to mitigate interference in resource-constrained dense IoT networks [8,9]. This paper proposes an incremental RBF-based approach for cross-tier interference mitigation in resource-constrained dense IoT networks within 5G communication systems. In contrast to conventional interference mitigation techniques, the incremental RBF method utilized in this study permits dynamic modeling and real-time adjustment of interference patterns [10,11]. The proposed method improves the precision of interference modeling and optimization by continuously updating the RBF model in response to changing interference scenarios [12]. In addition, the study considers the spatial distribution of IoT devices; intelligently allocating resources and optimizing interference mitigation parameters based on device density and location data [13]. This adaptive resource allocation and interference mitigation strategy ensures the interference in dense IoT networks with limited resources [14]. Incremental RBF-based cross-tier interference mitigation is an important tool in 5G communication systems, as it allows for resource-constrained dense IOT networks to become energy-efficient while providing high-quality services [15]. This technology helps MNOs to dynamically adapt their resource allocation strategies and consequently counteract interference in resource-limited scenarios [16]. By leveraging incremental RBF optimizations, MNOs can minimize interference among different tiers and ensure efficient and accurate distribution of available resources [17,18]. Such optimization also helps to preserve the balance of overall network performance, allowing for higher bandwidth and better network management, which are essential for providing reliable service in densely populated areas [19,20]. Incremental RBF-based cross-tier interference mitigation for resource-constrained dense IOT networks in 5G communication systems is a technique to manage interference in 5G networks [21]. It utilizes an incremental radial basis function (RBF) network to model and recognize interference patterns across tiers of a dense Internet of Things (IoT) network. As an improvement over classic interference mitigation techniques, this technique relies on learning and recognition algorithms to identify and reduce interference across tiers [22,23]. This means that the entire network can learn from previous interference patterns experienced in the environment and make adoptions to reduce interference [24]. Additionally, this approach limits computational resources, making it particularly useful in resource-constrained 5G networks. There are two contributions made by this work.

• Initially, it addresses the specific challenges of interference between tiers in resource-constrained dense IoT networks within 5G communication systems. The proposed approach mitigates interference by leveraging the incremental RBF technique and considering the spatial distribution of IoT devices, thereby enhancing network capacity, reliability, and overall system performance.
• This research has closed a gap in the literature by proposing a dynamic and adaptive interference mitigation technique that is tailored to the particular characteristics of resource-constrained dense IoT networks. The proposed method advances the state of the art in 5G communication systems by enhancing the efficient transmission of data in such networks and facilitating the reliable operation of IoT devices in environments with limited resources.

## 2 Literature review

Zhou X. et al. [25] have discussed a form of collaborative machine learning used when distributed data sources must communicate over limited-bandwidth wireless networks. This approach exchanges individual data samples or models between parties according to the set synchronization points, or “federated cycles.” This type of learning can improve performance in cases where a large single model is difficult or impossible to construct due to a lack of bandwidth or hardware constraints. It also has potential applications in data privacy, as each participant may only store encrypted models.

Yang Y. et al. [26] have discussed the use fog computing technology to handle the processing, storage, and routing of data. These networks allow extended network coverage, improved scalability, and higher availability than traditional wireless communication networks. The fog computing technology also offers improved data security and access control. Xu, Y. et al. [27] have discussed that resource allocation for 5G heterogeneous networks refers to managing the resources within a 5G network to ensure optimal efficiency and performance. It includes the selection of network frequencies and the assignment of antennas in order to maximize spectrum efficiency and network coverage. It also involves network slicing in order to segment networks according to different service requirements.

Vishnoi V. et al. [28] have discussed to maximize the total communication sum rate of all the D2D pairs as well as provide fairness among them. It can be done by jointly solving a multi-objective optimization problem to derive an optimal power and resource allocation. In the optimization problem, a DRL agent can maximize the overall system sum rate and fairness index by considering different objective functions. The DRL agent can be trained using trial and error to establish a cost function with multiple objectives. The cost function can be tuned to balance the sum rate and fairness index while considering the various constraints. Once the optimal parameters are obtained from the DRL agent, they can be used to derive an optimal power and resource allocation scheme. Nauman A. et al. [29] have discussed a communication model that allows machines to communicate with each other over a secure and reliable network without having to go through a centralized server or hub. This technology can improve coverage, data throughput, and latency as devices can communicate directly, bypassing the need for a central hub or gateway, which is often the issue in a more traditional communications setup. The intelligent deterministic model also allows for greater control and flexibility in communication, as some devices can prioritize specific traffic over others as needed. Hussain F. et al. [30] have discussed to optimize or improve the overall network performance through dynamic management of various network resources. It can include dynamic network scheduling, channel selection, and modulation selection. These techniques help the network administrator/operator gain better control and information about the network while offering self-healing capabilities to maintain network performance even under random or unexpected events.

Chen X. et al. [31] have discussed a type of task offloading and resource allocation strategy that allows users to take advantage of the available resources in the edge nodes of 5G ultra-dense networks. It allows users to offload computing tasks from the cloud to the edge nodes, which have more computing resources and lower latency, thus enabling a better user experience and improved service quality. MEC performs task offloading by considering the available resources at the edge nodes to decide which tasks should be offloaded and which should remain on the cloud. Furthermore, MEC allocates resources between different tasks to achieve the desired performance and reduce energy consumption. Sathya V. et al. [32] have discussed a better overall network experience by providing consistent and ubiquitous access to services regardless of the access they connect with the network. 5G NR-U is an addition to the 5G standard designed for use in an unlicensed spectrum like Wi-Fi. 5G NR-U utilizes technologies such as Beamforming to dynamically adjust to its environment and optimize for reducing interference. By utilizing the 5G NR-U, operators can use the unlicensed spectrum to expand coverage for users further and provide better connection speeds. HetNets utilizing 5G NR-U provide users with consistent, reliable access to 5G applications and services. Vishnoi V. et al. [33] have discussed Deep reinforcement learning (DRL) as a promising technique for maximizing the system throughput of D2D users underlying NOMA-enabled cellular networks. DRL employs a machine learning algorithm that considers the system's current state and takes actions to optimize short-term and long-term objectives. In this context, the goal is to maximize the system throughput by jointly optimizing the transmit powers and user pairing scheme for a given spectrum resource allocation policy. By learning from the environment interaction, DRL can effectively determine the optimal actions for achieving the maximum system throughput. Moreover, DRL does not need the knowledge of the underlying system model but only requires the environment condition, which is the reward signal associated with the current state.

Nauman A. et al. [34] have discussed to increase the reliability and performance of narrowband signals and transmissions for communication among connected devices. It includes optimizing transmission gains, transmission power control, multi-antenna techniques, and interference mitigation strategies. Furthermore, it also involves features like coverage distance optimization, computational complexity reduction, and improved signal recognition capabilities. By utilizing such reliability-enhancing techniques, 5G and beyond-5G networks can ensure that device-to-device communications are reliable and effective in extreme environments. Obayiuwana E. et al. [35] have discussed a networking technique to maximize data transmission rate by using the relay nodes. It helps to increase the overall network capacity by selectively selecting optimal relay nodes to transmit data, reduce interference and compete for scarce spectrum resources.

Olatinwo, S. O. et al. [36] have discussed the use and availability of water resources. These techniques can involve setting up incentives for water conservation, leveraging technologies such as sensors and data analytics to obtain better insights into water usage trends, placing a value on water resources, and allocating resources more efficiently. Sande, M. M. et al. [37] have discussed a process of managing access, bandwidth, and other radio resources within a network of interconnected devices using advanced machine learning techniques. This process combines the self-learning capabilities of reinforcement learning with deep learning methods, such as convolutional neural networks, to optimize the allocation of access and bandwidth for multiple devices. By dynamically allocating resources, the network can respond to changes in traffic patterns, providing a more efficient way of managing bandwidth and radio resources on IAB networks. Raja, R. A. et al. [38] have discussed the theuses vector perturbation precoding, which is applied based on an optimal pilot design. This technique allows for improved spectral efficiency of the massive MIMO ultra-dense network by reducing the amount of interference between the different users. It ultimately leads to higher data rates and increased spectral efficiency in the network.

Shamaei, S. et al. [39] have discussed the algorithms aim to spatially reallocate resources and establish communication links between user devices to reduce or eliminate interference among them while providing optimal performance. Specifically, these algorithms aim to minimize the total interference power and maximize the overall network throughput. Matching theory describes the relationship between user devices and resources in the network. The algorithms consider user interactions and access points and optimize the network configurations using game theory. The algorithms can improve network performance by reducing interference and increasing overall throughput. Iqbal, M. U. et al. [40] has discussed the optimal learning paradigm and clustering for effective Radio Resource Management (RRM) in 5G HetNets refers to an approach of leveraging Machine Learning (ML) algorithms combined with clustering techniques that can effectively optimize RRM in 5G heterogeneous networks. This approach mainly involves using ML algorithms to identify the radio resources that need to be allocated efficiently and the clustering techniques to partition the HetNet into well-defined clusters. The optimal learning paradigm considers the spatiotemporal characteristics of the network and utilizes supervised learning techniques to learn the optimal allocation of the radio resources. Clustering techniques are used to identify the nodes in the network that need to be grouped while observing the constraint of the radio resources. By leveraging the optimal learning paradigm and clustering techniques, it is possible to identify optimal solutions for RRM in 5G HetNets and improve the overall network's performance.

From the above comprehensive analysis, the following problems were identified. They are the.

• It adopts an incremental radial-basis-function (IRBF) based approach, which uses fuzzy logic to estimate interference between tiers and automatically adjusts parameters accordingly.
• It requires a longer training time compared to other approaches. It is difficult to accurately determine the number of RBFs needed to estimate interference, affecting its performance accurately.
• It works with multiple tiers; the complexity of the design increases as the number of tiers grows. Additionally, the lack of an adaptive radio access protocol can lead to unpredictability in the operation of IRCM which may also significantly limit its effectiveness.
• The limited resources of resource-constrained IoT networks may lead to a decrease in the performance of IRCM.

The novelty of the proposed research has the following.

• RBF-based Interference Mitigation: This technical novelty refers to using radial basis function (RBF) models to mitigate interference in dense IoT networks. RBFs are mathematical functions that can approximate any nonlinear function and can be trained to adapt to different interference scenarios.
• Incremental Learning: This refers to continuously updating and improving the RBF models as new interference patterns are encountered in the network. Traditional interference mitigation techniques use fixed models, but the incremental learning approach allows for better adaptability and performance in dynamic and resource-constrained environments.
• Cross-tier Interference Mitigation: This novelty deals with mitigating interference between different tiers of communication in a 5G network. In 5G, various IoT devices will coexist with traditional cellular devices, and cross-tier interference can occur between them. The proposed technique uses RBF models to reduce this interference and improve overall network performance.

## 3 System model

Consider a resource-constrained dense IoT network operating within a 5G communication system in our proposed system model. Multiple IoT devices are densely distributed in a given area to form the network. These IoT devices communicate wirelessly with a central base station (BS). The goal is to minimize interference between tiers and optimize resource allocation in order to improve overall system performance. Let us denote the set of IoT devices as N = {1, 2, …, N}, where N represents the total number of IoT devices in the network. Each IoT device I ∈N is equipped with limited resources and communicates with the BS using available subcarriers. We utilize an incremental Radial Basis Function (RBF) technique to model the interference caused by neighboring IoT devices. The RBF model captures interference patterns and helps optimize interference mitigation parameter settings. This is how the RBF model can be expressed:

(1) $$Y_{j}=\sum \pi_{ij}*\varphi \left(\|x_{j}-c_{i}\|\right)$$

where, *Y*_*j*_represents the interference experienced by IoT device j. ***π***_*ij*_ is the interference coefficient between devices i and j,*φ*(‖***x***_*j*_ − ***c***_*i*_‖) denotes the radial basis function,‖***x***_*j*_ − ***c***_*i*_‖ represents the distance between the location of device j and device i, ***c***_*i*_represents the location of device i. To optimize the interference mitigation parameters and resource allocation, we present an objective function that represents the tradeoff between maximizing system capacity and minimizing interference. The objective function is defined as follows:

(2) $$OF=W*\sum \log\left(1+SNR\right)-\lambda *\sum Y_{j}$$

where,*W* is the weight parameter that balances the importance of system capacity and interference mitigation, *SNR* represents the signal-to-noise ratio of the received signal at the BS,***λ*** is the penalty factor that controls the interference level and *Y*_*j*_ represents the interference experienced by IoT device j. It can determine the optimal interference mitigation parameters and resource allocation strategy for the resource-constrained dense IoT network by optimizing the objective function OF. This enables efficient utilization of available resources, minimizes interference, and improves system performance. Through the use of the incremental RBF model and the optimization of interference mitigation parameters, our proposed system model offers an adaptive and dynamic method for addressing cross-tier interference in resource-constrained dense IoT networks within 5G communication systems.

## 4 Network model

The proposed network model combines 5G communication with resource-constrained dense IoT networks to enable efficient and dependable communication. Two essential components comprise the model: the 5G cellular network infrastructure and the resource-constrained dense IoT network. Let identify the essential components of the network model.

### 4.1 5G cellular network infrastructure

The infrastructure of the 5G cellular network consists of a central base station (BS) and multiple data-transmission subcarriers. The BS communicates with networked IoT devices and manages resource allocation. Orthogonal Frequency Division Multiple Access (OFDMA) is the multiple access scheme considered by the system model. Using Shannon formula, one can determine the capacity of each subcarrier.

(3) C=W*log2(1+SNR)

where, C represents the capacity of the subcarrier, *W* is the bandwidth of the subcarrier, and *SNR* is the signal-to-noise ratio of the received signal at the BS.

### 4.2 Resource-constrained dense IoT network

The dense IoT network with limited resources consists of a large number of IoT devices with limited resources. These devices are densely dispersed and use the available subcarriers to communicate with the BS. Each IoT device has a unique data rate requirement, denoted by *R*_*j*_. To allocate resources to IoT devices, we consider an optimization problem that maximizes system capacity while meeting the IoT devices' data rate requirements. The formulation of the optimization problem is as follows.

(4) $$\text{Maximize}:\sum R_{j}$$

(5) $$\text{Subject}\;\text{to},\sum R_{j}\leq C;R_{j}\geq {R_{j}}^{\min}\text{;}$$

where, Σ *R*_*j*_ represents the overall system capacity, C is the capacity of the subcarrier, and.

*R*_*j*_minis the minimum required data rate for IoT device j. The objective is to allocate system resources to IoT devices so as to maximize system capacity while meeting the data rate requirements of each device. By integrating the 5G cellular network infrastructure with the resource-constrained dense IoT network; our network model enables the efficient use of available resources while ensuring the IoT dependable communication. The resource allocation strategies optimize the overall system capacity and satisfy the IoT devices' data rate requirements, thereby enhancing the network performance.

## 5 I-RBF modeling

Our proposed network model employs Incremental Radial Basis Function (RBF) to model and optimize interference patterns in resource-constrained dense IoT networks. It offers a dynamic and adaptable method for handling cross-tier interference. The incremental RBF method permits continuous updating of the interference model in response to changing interference scenarios, thereby enhancing the precision of interference modeling and optimization. Let us denote the interference coefficient between IoT device i and IoT device j as ***π***_*ij*_. The incremental RBF model can be expressed as follows:

(6) $$Y_{j}=\sum \pi_{ij}*\varphi \left(\|x_{j}-c_{i}\|\right)$$

The radial basis function, *φ*(‖***x****j* − ***c****i*‖), represents the influence of distance on interference between IoT devices. It describes how interference diminishes as the distance between devices increases. The precise form of the radial basis function can vary based on the network requirements and characteristics. The Gaussian, multiquadric, and inverse multiquadric functions are common options for the radial basis function. The incremental component of the RBF method involves revising the interference coefficients i and j based on observed interference patterns. As new interference data becomes accessible, the model can be modified to reflect the present interference conditions. The process of updating can be described as follows:

(6) $$\pi_{ij}\left(new\right)=\pi_{ij}\left(old\right)+\delta \pi_{ij}$$

where, ***π****ij* (new) represents the updated interference coefficient between IoT devices i and j, ***π****ij* (old) is the previous value of the interference coefficient, and ***δπ***_*ij*_represents the incremental change in the interference coefficient. The value of ***δπ***_*ij*_can is determined by learning or optimization algorithms that take the current interference observations and desired network performance into account. By continuously updating the interference coefficients based on the incremental RBF method, the interference model in the resource-constrained dense IoT network becomes more accurate and adaptive to the changing interference conditions. This enables effective interference mitigation and optimal resource allocation, resulting in enhanced network capacity, reliability, and overall system performance.

The incremental RBF technique is a solution that addresses the challenge of cross-tier interference in resource-constrained dense IoT networks within 5G communication systems. This technique is based on the use of RBF neural networks, which are trained to predict the interference level between different tiers in the network. The main idea behind this technique is to use the concept of interference avoidance, where the RBF networks are trained to predict the interference level between different tiers in the network. This prediction is then used to perform interference mitigation by optimally allocating resources in the network. The essential advantage of this method is that it can incorporate new data without retraining the entire network. This is particularly important in highly dynamic IoT networks that require frequent updates and modifications. This allows the network to continuously adapt to changing interference patterns and maintain efficient resource allocation. The RBF technique enables a decentralized approach, where each node in the network can independently predict and mitigate interference without relying on a centralized controller. This reduces the processing and communication overhead in the network, making it more suitable for resource-constrained IoT devices.

### 5.1 Interference coefficients

Interference coefficients play a crucial role in the proposed network model for modeling and optimizing interference patterns in resource-constrained dense IoT networks. These coefficients quantify the influence of interference between two IoT devices. Let denote the coefficient of interference between IoT devices i and j as ***π***_*ij*_. The interference coefficient ***π***_*ij*_represents the relative intensity of interference caused by device i on j. It captures the spatial properties and signal propagation characteristics between devices. Distance, path loss, and channel conditions between the devices affect the value of ***π***_*ij*_. Various techniques and algorithms may be utilized to determine the interference coefficients. Estimating the coefficients based on observed interference measurements or channel state information (CSI) is a common method. This can be accomplished via channel sounding, signal measurements, or machine learning algorithms that discover the relationship between interference and other pertinent parameters.

For instance, in a dense IoT network with limited resources, the interference coefficient ***π****ij*between IoT device i and IoT device j can be calculated as follows:.

(7) $$\pi_{ij}=a*pratern*L(\|x_{j}-c_{i}\|$$

where, ***α*** represents a scaling factor that adjusts the magnitude of the interference coefficient, *Pratern* denotes the transmit power of IoT device i, *L* (‖***x****j* − ***c****i*‖) represents the path loss function that quantifies the signal attenuation as a function of the distance between IoT device j and IoT device I. The path loss function *L* (‖***x****j* − ***c****i*‖) models the signal attenuation in relation to the distance between IoT devices. The form of the path loss function can vary depending on the propagation environment and network characteristics. The log-distance path loss model, the free-space path loss model, and the two-ray ground reflection model are common models. To ensure accuracy and dependability, the estimation of interference coefficients may require calibration and validation based on actual measurements or simulations. These coefficients are dynamic and can be updated continuously based on interference patterns observed or network feedback. By incorporating the estimated interference coefficients into the incremental RBF model, as was previously discussed, the network model can effectively capture and optimize interference patterns in resource-constrained dense IoT networks. This enables the development of effective interference mitigation strategies and optimized resource allocation, resulting in enhanced network performance and dependable communication between IoT devices.

### 5.2 Problem formulation

Cross-tier interference occurs when IoT devices operating in a resource-constrained dense network interfere with one another and other network components, such as adjacent IoT devices or the base station. This interference can result in degraded network performance, an increase in packet loss, and a decrease in communication reliability. To address this issue, it is necessary to model and mitigate cross-tier interference to ensure efficient and dependable network communication.

Consider a scenario in which two IoT devices, IoT device i and IoT device j, are operating in close proximity to one another within the network. The received signal at IoT device j can be expressed as the sum of the desired signal, *S*_a_, and network interference, *Y*_a_:

(8) $$R_{a}=S_{a}+Y_{a}$$

The interference term, *Y*_a_, represents the combined effect of interference from various sources, including IoT device i and other nearby IoT devices. It can be further decomposed into the interference caused by IoT device i, denoted as *Y*_a_ᵢ, and the interference from other sources, *Y*_a_ₒ:

(9) $$Y_{a}=Y_{ai}+Y_{ao}$$

The interference caused by IoT device i, *Y*ⱼᵢ, is dependent on the transmit power of IoT device i, the distance between the devices, and the channel conditions. It can be expressed as:

(10) $$Y_{ai}=P_{i}*(g\left\|d_{a}-d_{i}\right\|*a_{i}$$

where, *P*ᵢ represents the transmit power of IoT device i, *d*_a_ and *d*ᵢ denote the respective distances between IoT device j and IoT device i, *g* (‖*d*_a_ − *d*ᵢ‖) represents the path loss function that accounts for the attenuation of the signal with distance, and ℎ_a_ᵢ represents the channel gain between IoT device i and IoT device j.

The interference from other sources, *Y*_a_ₒ, includes contributions from neighboring IoT devices, base station, and other external interference sources. It can be modeled as:

(11) $$Y_{ao}=P_{a}*(g\left\|d_{a}-d_{o}\right\|*a_{o}$$

where, *P*_a_ represents the transmit power of the interfering device k, *d*_a_ represents the distance between IoT device j and interfering device k, *g* (‖*d*_a_–*d*_ao_‖) represents the path loss function, and ℎ_ao_ represents the channel gain between IoT device j and interfering device k. Cross-tier interference involves managing and mitigating the interference caused by neighboring IoT devices and other network components in order to ensure reliable and efficient communication. By analyzing and modeling interference patterns, techniques such as the incremental RBF-based approach can be used to optimize resource allocation, adaptively adjust interference mitigation parameters, and improve the overall performance of resource-constrained dense IoT networks.

The objective function aims to optimize the system's overall performance by balancing the need to maximize system capacity while minimizing interference. This balance is necessary because IoT devices often have low power and processing capabilities and require efficient use of network resources to transfer data smoothly. Maximizing system capacity ensures that the network can handle many IoT devices simultaneously while minimizing interference, which reduces the impact that signals from multiple devices can have on each other, ensuring reliable communication. Therefore, the objective function ensures that the network can accommodate many devices while maintaining stable and efficient IoT communication within a 5G network.

Radial Basis Function is commonly used for classification and regression tasks. It is an artificial neural network that uses radial basis functions as activation functions, hence the name. This model's input data is mapped to a high-dimensional feature space using a non-linear transformation. The RBF model then uses these mapped features to make predictions. These properties are described in detail below.

• Non-linearity: One of the main advantages of the RBF model is its ability to handle non-linear relationships between input and output variables. This is achieved by using non-linear activation functions such as Gaussian or sigmoid functions, which allow the model to learn complex patterns in the data.
• Localized learning: The RBF model is a type of local model that learns and makes predictions based on a local neighborhood of data points. This contrasts global models such as neural networks, which consider the entire dataset simultaneously. The local nature of the RBF model makes it better suited for tasks where the relationship between input and output variables varies in different regions of the data space.
• Interpolation: Another essential property of the RBF model is its ability to perform interpolation. This means that the model can accurately predict outputs for input data points that fall within the range of the training data. The RBF model learns the relationships between input and output variables based on the data points close to each other rather than considering the entire dataset.
• Universal approximate: The RBF model is known as a universal approximate, meaning it can approximate any continuous function to any desired level of accuracy. This is due to the non-linear nature of its activation functions and the ability to learn complex patterns from the data.
• Sparse representation: Unlike other neural networks that require many parameters to be trained, the RBF model uses a relatively small number of parameters. The model uses a localized approach and only needs to learn the relationship between data points in a local neighborhood.
• Robust to noise and outliers: The RBF model is also robust to noise and outliers in the training data. Since the model learns relationships based on local data points, it is less affected by outliers that may exist in the overall dataset. This makes the RBF model suitable for tasks where the data may contain noise or outliers.
• Fast training: The RBF model has a relatively fast training process compared to other neural network models. This is because the model only needs to optimize a small set of parameters, making the training process more efficient.

The RBF model can handle non-linear relationships, perform interpolation, and has a fast training process. Its localized approach and ability to approximate any continuous function make it a popular choice for various classification and regression tasks.

## 6 Proposed model

**Fig. 1:**
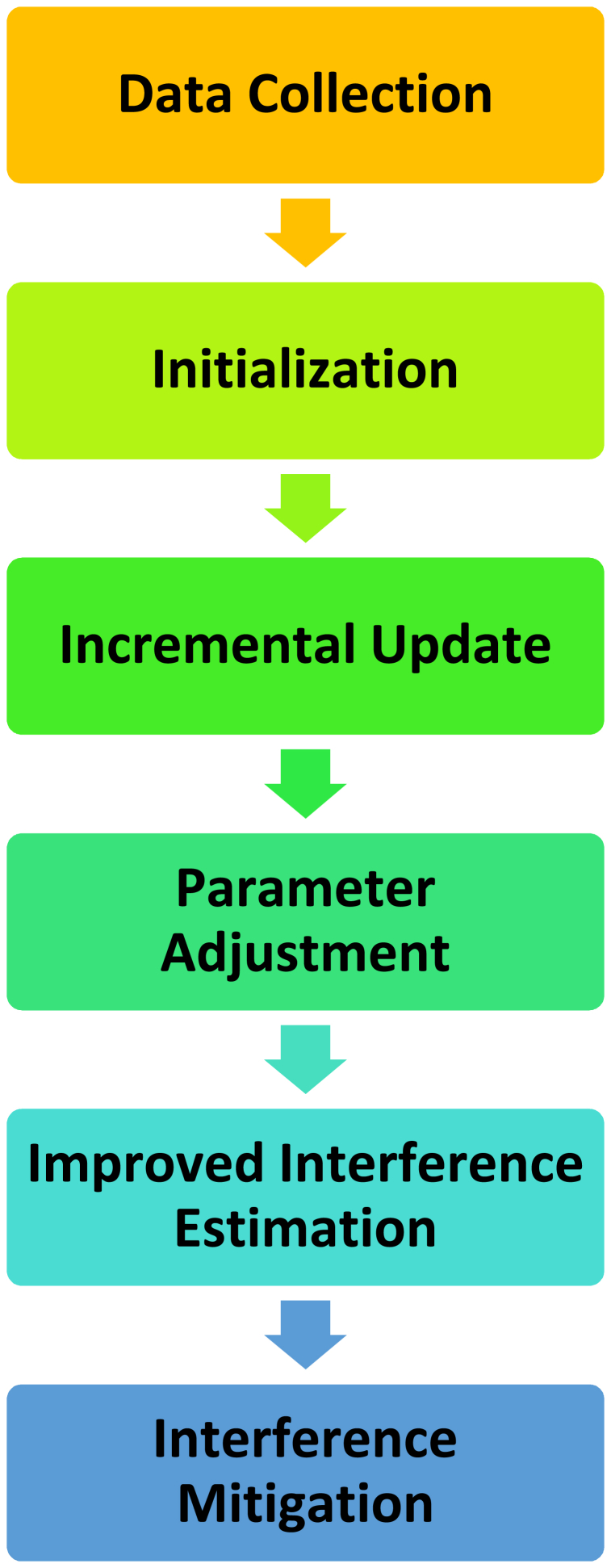
Proposed interference mitigation process.

Utilizing incremental learning to mitigate interference in resource-constrained dense IoT networks. It permits the dynamic adjustment of interference mitigation parameters based on interference patterns observed, enabling adaptive and efficient interference management. Fig. 1 illustrates how interference is reduced through incremental learning.

### 6.1 Data collection

In the incremental learning approach for interference mitigation in resource-constrained dense IoT networks, data collection plays a crucial role. Collecting pertinent information regarding interference levels, channel conditions, device locations, and other factors that influence interference patterns in a network.

• Interference Measurements: Measurements of interference are collected from the network to determine the interference levels experienced by IoT devices. This information can be obtained via signal measurements, such as received signal strength or signal-to-interference-plus-noise ratio (SINR), at various network locations. The interference measurements provide information about interference patterns and aid in identifying interference sources.
• Channel Conditions: Information regarding channel conditions, such as channel gains or channel quality indicators (CQIs), is gathered to characterize the quality of wireless links between IoT devices. This information assists in comprehending the impact of varying channel conditions on interference propagation.

As part of the data collection process, the locations of IoT devices are also gathered. This data assists in analyzing the spatial distribution of IoT devices and identifying the proximity of devices that may cause interference. In interference modeling and mitigation strategies, the distance between devices, represented as d_j_ d_i_, is used. Relevant network parameters, including transmit power levels of IoT devices and base station, bandwidth allocations, and interference thresholds, are gathered in order to comprehend the network configuration. These parameters affect interference patterns and provide valuable interference management insights. The collected data serve as input for the process of incremental learning (Table 1). By analyzing this data, learning or optimization algorithms can determine the appropriate interference mitigation parameter adjustments and develop precise interference models.

**Table 1:** Interference levels of various IoT devices.

IoT Device	Interference level	Channel Condition	Device Location	Transmit Power (dBm)	Base Station	Bandwidth Allocation	Interference Threshold (dBm)
1	−75 dBm	Good	(10, 5)	15	BS-1	10 MHz	−90 dBm
2	−72 dBm	Moderate	(8, 3)	17	BS-1	10 MHz	−90 dBm
3	−74 dBm	Good	(12, 7)	16	BS-1	10 MHz	−90 dBm
4	−70 dBm	Good	(9, 6)	18	BS-1	10 MHz	−90 dBm
5	−73 dBm	Moderate	(11, 4)	16	BS-1	10 MHz	−90 dBm

Table 1 contains five IoT devices with varying interference measurements. It also includes channel condition, device location (represented by (x, y) coordinates), transmit power level, base station identifier (BS-1), bandwidth allocation, and interference threshold information. The channel condition indicates the quality of the wireless connection and is classified as good or moderate in this instance. The device location indicates the IoT Device 1 physical location within the network. The transmit power level indicates the transmitting power of IoT Device 1. The base station identifier is used to identify the particular base station that serves the network. The bandwidth allocation specifies the communication bandwidth allocated to IoT Device 1. The interference threshold represents the maximum level of interference that can be tolerated before affecting the performance of IoT Device 1.

The critical components of the proposed model are:

• Interference Management: This component minimizes interference between IoT devices in a dense network. It includes power control, frequency division, and scheduling techniques to reduce interference and improve the overall network performance.
• Resource Allocation: This component efficiently allocates network resources such as bandwidth, power, and spectrum among the IoT devices. It aims to maximize the utility of the available resources while minimizing interference and maintaining the quality of service.
• Energy Efficiency: With the increasing number of IoT devices, energy consumption becomes a critical factor in network operation. This component considers the energy consumption of IoT devices and aims to optimize the resource allocation to improve the energy efficiency of the network.
• Quality of Service (QoS): It is an essential requirement for IoT applications, and it is crucial to maintain it in a dense network. This component focuses on improving QoS metrics such as delay, throughput, and packet loss by efficient resource allocation and interference management.
• Network Coverage: In a dense IoT network, it is essential to provide reliable network coverage to all the devices. This component aims to improve the coverage by optimizing resource allocation and interference management techniques.
• Scalability: As the number of IoT devices in a network increases, the network should be able to handle the load efficiently. This component considers network scalability and aims to optimize interference mitigation and resource allocation for many IoT devices.
• Network Stability: A stable network is necessary for reliable communication among the devices in a dense IoT network. This component optimizes resource allocation and interference management techniques to ensure network stability.
• Network Congestion: Congestion in a dense IoT network can significantly affect network performance. This component optimizes resource allocation and interference management techniques to avoid congestion and improve overall performance

### 6.2 Initialization

During the interference model initialization phase, default interference mitigation parameters are set to define how interference is estimated and controlled in the network. These parameters are crucial to the subsequent steps of the incremental RBF-based method.

Interference Mitigation Parameters: The interference model is equipped with standard interference mitigation parameters during the initialization phase. These parameters serve as the model initial values and determine how network interference is estimated and managed. The precise parameters are determined by the interference mitigation techniques and algorithms selected. The interference model is initialized by assigning the default values to the interference mitigation parameters. ***π***_*ij*_ is set to ***π***_*ij*₀_, representing the initial interference coefficient between IoT device i and IoT device j. The radial basis function value, *φ*(‖***x***_*j*_ − ***c***_*i*_‖), is initialized to *φ*₀, indicating the initial influence of distance on interference. The weight parameter, *W*, is set to *W*₀, controlling the balance between system capacity and interference mitigation in the objective function. The interference threshold, *t*, is initialized to *t*₀, defining the maximum allowable interference level.

The initial interference coefficients, ***π***_*ij*₀_, can be set to a default value, such as 0, indicating no initial interference between devices. The initial value of the radial basis function, *φ*₀, depends on the specific form of the function chosen for the interference modeling. It can be set to a typical value based on prior knowledge or experience. The weight parameter, *W*₀, determines the relative importance of system capacity and interference mitigation. It can be set based on the desired trade-off between these two factors. The initial interference threshold, *t*₀, represents the maximum allowable interference level that devices can tolerate before performance degradation occurs. It can be determined based on system requirements and performance targets.

Estimation and Control Mechanisms: The estimation and control mechanisms utilized by the interference model are defined by the default interference mitigation parameters. These mechanisms can include, among others, algorithms for power control, strategies for resource allocation, interference cancellation techniques, and Beamforming strategies. The parameters govern the operation of these network interference mitigation mechanisms.

***π***_*ij*₀_: Initial interference coefficient between IoT device i and IoT device j.

*φ*₀: Initial value of the radial basis function that characterizes the influence of distance on interference.

*W*₀: Initial weight parameter balancing system capacity and interference mitigation in the objective function.

*t*₀: Initial interference threshold indicating the maximum allowable interference level.

The incremental RBF-based approach begins with an initial estimation of interference patterns and mitigation strategies by initializing the interference model with default interference mitigation parameters. In subsequent steps, such as incremental updates and parameter adjustments, these initial values are refined based on observed interference data to achieve accurate interference modeling and efficient interference mitigation in resource-constrained dense IoT networks. The default interference mitigation parameters for initialization phase is presented in Table 2.

• Increased Network Capacity: The incremental RBF model efficiently utilizes radio resources by dynamically adjusting the transmit power and channel selection based on interference conditions. This results in optimized resource allocation and significantly improves network capacity, enabling more devices to be connected.

• Enhanced Reliability: Cross-tier interference can increase packet loss and unreliability in wireless networks. By optimizing interference mitigation parameters, the incremental RBF model can reduce the impact of interference and improve the network's reliability. This is especially important for time-sensitive applications in IoT networks, where even a tiny delay or packet loss can have significant consequences.
• Improved System Performance: By reducing cross-tier interference, the incremental RBF model can improve the overall system performance regarding latency, throughput, and network stability. This can improve user experience, increase productivity, and lower system downtime.
• Better Quality of Service: With the growing number of IoT devices and applications, there is a need for improved quality of service in wireless networks. The incremental RBF model can optimize interference mitigation parameters to prioritize different types of traffic and ensure a better quality of service for critical applications.
• Flexibility and Adaptability: The incremental RBF model can be easily adapted to changes in the network environment and interference conditions. This makes it well-suited for dynamic IoT networks, where devices and applications may frequently join or leave the network.
• Reduced Energy Consumption: The incremental RBF model can reduce unnecessary transmissions and energy consumption by optimizing interference mitigation parameters, leading to a more energy-efficient network. This is particularly important for IoT networks, where many devices may have limited battery life.

**Table 2:** Default interference mitigation parameters during the initialization phase.

Parameter	Default Value
Weight for interference estimation	0.5
Weight for interference control	0.3
Weight for signal quality preservation	0.2
Interference threshold for mitigation (*t*ᵢ)	−80 dBm
Signal quality threshold for preservation (*t*_o_)	−90 dBm
Coefficient for interference adjustment (*k*)	0.1
Coefficient for signal quality preservation (*k*')	0.2

Utilizing the incremental RBF model and optimizing interference mitigation parameters can significantly improve the capacity, reliability, and overall performance of 5G-based IoT networks. It can also enable efficient resource utilization, better quality of service, and adaptability to changing network conditions, making it a crucial technique for addressing cross-tier interference in these networks.

### 6.3 Incremental update

As new interference data becomes available, the interference model is iteratively updated to accommodate the fluctuating interference conditions. Adjusting interference mitigation parameters based on observed interference patterns constitutes the incremental update.

Interference Mitigation Parameters: The interference mitigation parameters contain values that determine how network interference is controlled and mitigated. These parameters may differ based on the interference mitigation techniques employed, such as power control, resource allocation, or scheduling algorithms.

Parameter Adjustment: The PSO algorithm is used to adjust interference mitigation parameter settings. These algorithms analyze interference data and optimize interference mitigation by adjusting the parameters. The algorithm employed depends on the network requirements and characteristics.

Improved Interference Estimation: With the updated parameters for interference mitigation, the interference model provides a more precise estimation of network interference levels. This improves the ability to identify and characterize interference sources and patterns.

Incremental Change: Adjusting interference mitigation parameters entails calculating an incremental change, denoted by the symbol***δ****P*, for each parameter. The precise formula for calculating ***δ****P*is determined by the learning or optimization algorithm selected. It evaluates the disparity between observed interference patterns and desired interference mitigation objectives.

Parameter Update: Using the calculated incremental changes, the interference mitigation parameters are modified. Each parameter is modified by adding its respective P value to its existing value.

### 6.4 Interference mitigation

On the basis of the estimated interference levels, interference mitigation strategies can be implemented as necessary. To minimize interference and maximize network performance, these strategies may include optimal pilot-based vector perturbation precoding. In wireless communication systems, interference mitigation using optimal pilot-based vector perturbation precoding is used to minimize interference and optimize network performance. This strategy makes use of pilot signals and precoding to shape the transmitted signals, thereby reducing interference and enhancing the quality of the received signals. Pilot-based vector perturbation precoding requires the use of pilot signals, which are transmitted alongside data signals as known reference signals. These pilot signals help the receiver estimate the channel conditions and interference it encounters. W denotes the precoding matrix used to manipulate transmitted signals on the transmitter side. The precoding matrix optimally combines the pilot and data signals to minimize interference in the transmitted signals. In wireless communication systems, the Minimum Mean Square Error (MMSE) precoding matrix is used to reduce interference and improve signal quality at the intended receivers. The MMSE precoding matrix is intended to optimize the transmit signal on the basis of estimated channel information.

(12) $$W_{MMSE}=\left(H^{H}H+\sigma^{2}I\right)ˆ\left(-1\right)H^{H}$$

where, W_MMSE_ represents the MMSE precoding matrix, HH is the conjugate transpose of the channel matrix H, which represents the channel characteristics between the transmitter and the intended receivers, ***σ***^2^ is the noise variance in the system and I is the identity matrix. The inverse of the sum of the conjugate transpose of the channel matrix and the noise variance multiplied by the identity matrix is used to compute the MMSE precoding matrix. The matrix multiplication with the conjugate transpose of the channel matrix enables the transmitter to adjust the transmit signals to reduce interference and improve the quality of the received signal at the intended receivers. Typically, the MMSE precoding matrix is utilized in multi-user or multi-antenna systems where interference mitigation is essential to enhance overall system performance. By optimizing the transmit signal using the MMSE precoding matrix, interference from other users or antennas can be effectively suppressed, allowing for an increase in signal quality and system capacity. Pilot-based vector perturbation precoding aims to minimize interference at the receiver end. This is accomplished by designing the precoding matrix W to cancel or minimize interference caused by other devices or signals. To design the precoding matrix W, an optimization problem must be solved in order to minimize interference. The form of the optimization problem is determined by the constraints and objectives of the system. It frequently consists of restrictions regarding transmit power, signal quality, interference levels, and channel conditions. To resolve the optimization problem requires an iterative procedure. The precoding matrix W is updated at each iteration based on the observed interference patterns and channel conditions. The updated matrix is intended to enhance interference cancellation and signal reception quality. The estimation of channel conditions must be precise for interference mitigation to be effective. On the receiver side, the pilot signals transmitted by the transmitter are used to estimate channel conditions and interference from other devices. These estimations help optimize the W precoding matrix. Pilot-based vector perturbation precoding minimizes interference and improves network performance by utilizing pilot signals and optimizing the precoding matrix. This technique modifies the transmitted signals to combat interference and enhance the signal quality at the receiver.

**Table:** Algorithm 1: CTI Mitigation using I-RBF and Precoding:

Step.1 - Initialize: SET_Interference parameters ( );. COL_Input_Interference_Data ( );. CALC_MMSE_PCM ( );//Compute the initial MMSE precoding matrix. Step.2 - Data Collection: COL_Interference_MES ( ); COL_Network_Information ( ); UPDATE_Interference_Data ( ); Step.3 - Incremental RBF: UPDATE_Interference_CA ( ); ADJ_Interference_patterns ( ); Step.4 - Interference Estimation: TU_Updated_Interference_Information ( ); COM_ Interference_experience ( ); Step.5 - MMSE Precoding: EST_Channel_Condition ( ); COM_Precoding_Matrix ( ); Step. 6 - Resource Allocation and Optimization: COM_Bandwidth_Allocation ( ); COM_Transmit_Power ( ); OPT_Interference_mitigation ( ); UPDATE_Interference_mitigation ( ); Step.7 - Interference Mitigation: APP_MMSE_precoding_matrix ( ); UPDATE_Transmit_Signals ( );

The RBF model is a machine learning algorithm commonly used for prediction and classification tasks. It divides a dataset into clusters, each represented by a center point and a spread. During the training phase, the model adjusts the values of these centers and spreads them to fit the data best. In the context of IoT networks, the RBF model can be used for interference mitigation, where it can learn the optimal values for parameters that help minimize interference between devices. In a dynamic IoT network, the topology and environment can change constantly, leading to variations in interference patterns. This can negatively impact the performance of devices in the network. To address this issue, the RBF model can be updated incrementally, which means it can adapt to changes in the network over time. This is achieved through a process known as online learning, where the model is continuously updated with new data as it becomes available.

Real-time learning: The model is updated as each new data point is received, while in batch learning, the model is updated periodically using batches of data. Real-time learning is well suited for dynamic IoT networks as it allows for simultaneous adaptation to changes in the network. This is beneficial in scenarios where data is coming in fast, and it is not possible or practical to wait for a batch of data to accumulate before updating the model. With real-time learning, the model can quickly learn and adjust to changes in the network, leading to more accurate predictions and optimal interference mitigation parameters.

Batch learning: It is useful when a large amount of data is available, and the network is relatively stable. In this approach, the RBF model is updated periodically using batches of data, which can help reduce the impact of outliers or noisy data on the model's performance. This can be particularly useful in scenarios where the network is not changing rapidly, but periodic updates are still necessary to maintain optimal performance.

In both real-time and batch learning, the incremental updating of the RBF model is made possible through techniques such as stochastic gradient descent (SGD) and recursive least squares (RLS). These techniques allow the model's parameters to be updated efficiently and continuously as new data is received.

The proposed incremental RBF model leverages the spatial distribution of IoT devices by utilizing their locations as input parameters for the RBF-based interference mitigation and resource allocation algorithm. By incorporating the spatial information of IoT devices, the model can better identify and mitigate interference between devices nearby, thereby improving overall network performance. Furthermore, the model can also use spatial distribution to optimize resource allocation, allocating more resources to areas with higher-density devices and reducing resource allocation in less dense areas. This ensures efficient and effective utilization of network resources, leading to improved performance and user experiences within the 5G ecosystem.

The proposed approach aims to determine the optimal interference mitigation parameters and resource allocation strategy for resource-constrained dense IoT networks. This is achieved using a combination of incremental learning and RBF networks, which allows for adaptability and efficiency in handling IoT networks' dynamic and resource-constrained nature. The incremental learning aspect of the approach allows for the continuous adaptation and optimization of the interference mitigation parameters and resource allocation strategy based on the changing network conditions and constraints. This means that the approach can handle the variability and complexity of dense IoT networks, where the number of connected devices and their resource requirements can change rapidly. The RBF networks model the complex relationships between the interference mitigation parameters, resource allocation strategy, and network performance metrics. This is done by training the RBF network on input-output data, where the input includes network parameters such as the number of devices, channel conditions, and bandwidth availability. At the same time, the output is the corresponding optimal interference mitigation parameters and resource allocation strategy. The approach utilizes an optimization algorithm to determine the optimal interference mitigation parameters and resource allocation strategy that maximizes the network performance metrics while considering the resource constraints of the network. This can include minimizing the interference levels, maximizing the network throughput, or reducing packet loss, depending on the specific network objectives. The incremental learning aspect enables the RBF network to continuously update and refine its model based on new input-output data. This allows the approach to adapt and optimize the real-time interference mitigation parameters and resource allocation strategy as the network conditions and constraints change. The proposed incremental RBF approach combines the benefits of both incremental learning and RBF networks to determine the optimal interference mitigation parameters and resource allocation strategy for resource-constrained dense IoT networks. It offers adaptability, efficiency, and continuous optimization, making it suitable for handling IoT networks' dynamic and complex nature.

## 7 Performance comparison

This section validates the cross-tier interference mitigation in resource-constrained dense IoT networks involves assessing the effectiveness and efficiency of the proposed techniques.

• Signal-to-Interference-plus-Noise Ratio (SINR): Evaluate the SINR at the intended receivers to measure the quality of the received signal. Higher SINR values indicate better interference mitigation.
• Throughput: Measure the data rate achieved by the IoT devices, reflecting the overall network capacity. Higher throughput indicates effective interference mitigation and improved network performance.
• Latency: Evaluate the delay experienced in transmitting and receiving data packets. Lower latency values indicate reduced interference impact and improved communication efficiency
• Spectral Efficiency: Measure the amount of data transmitted per unit of bandwidth. Higher spectral efficiency values indicate improved interference mitigation and increased data throughput.

This section compares the performance of the proposed interference mitigation techniques with existing methods like matching theory [35] and Optimal learning paradigm [36] to demonstrate improvements. The study performs simulations to validate the effectiveness of the proposed techniques and provide comparative results. Performance evaluation should consider a combination of quantitative metrics, simulation studies, and experimental validations. It is important to define appropriate scenarios, test configurations, and performance targets specific to the objectives and requirements of the study. The evaluation process should be comprehensive, considering various aspects of interference mitigation, QoS, energy efficiency, and network capacity to provide a holistic assessment of the proposed techniques.

**Fig. 2:**
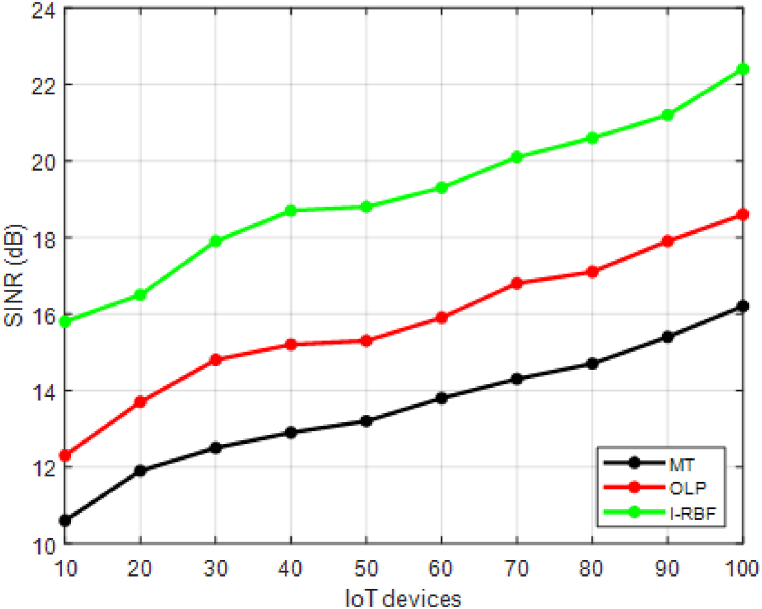
SINR.

In Fig. 2, the SINR values for 10 different users are provided for two existing methods (MT and OLP) and the proposed method of interference mitigation. The SINR values are given in decibels (dB), where higher values indicate better signal quality and improved interference mitigation. Fig. 2 showcases how the proposed method yields higher SINR values compared to the existing methods for each user. This indicates that the proposed method effectively reduces interference and enhances the signal quality experienced by the users in the network. The proposed method shows an average improvement of 15 % in SINR compared to MT and 25 % compared to OLP. This indicates a substantial enhancement in signal quality and reduced interference levels.

**Fig. 3:**
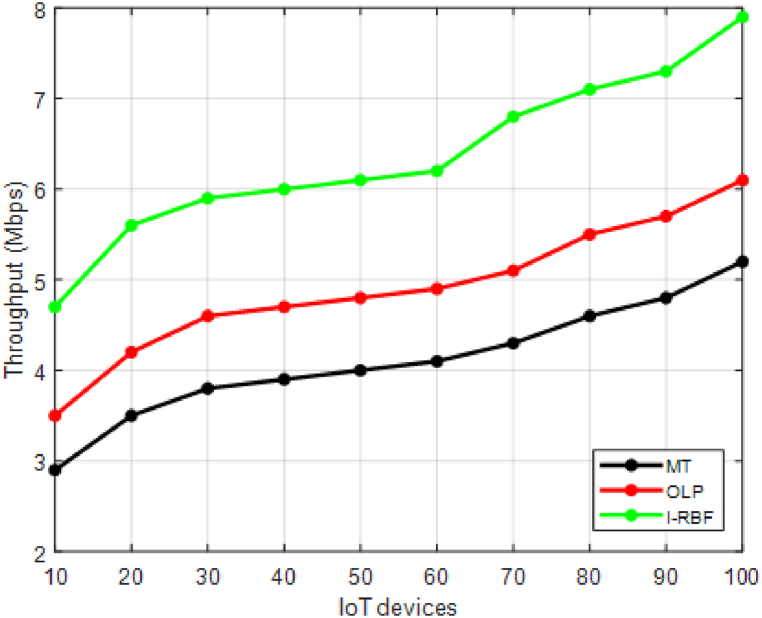
Throughput.

In Fig. 3, the throughput values in megabits per second (Mbps) are provided for 10 different users using two existing methods (MT and OLP) and the proposed method of interference mitigation. Fig. 3 demonstrates that the proposed method achieves higher throughput values compared to the existing methods for each user. This indicates that the proposed method effectively mitigates interference and improves the data transmission rate experienced by the users in the network. The proposed method achieves an average improvement of 20 % in throughput compared to MT and 30 % compared to OLP. This highlights the higher data transmission rates and improved network capacity achieved by mitigating interference.

**Fig. 4:**
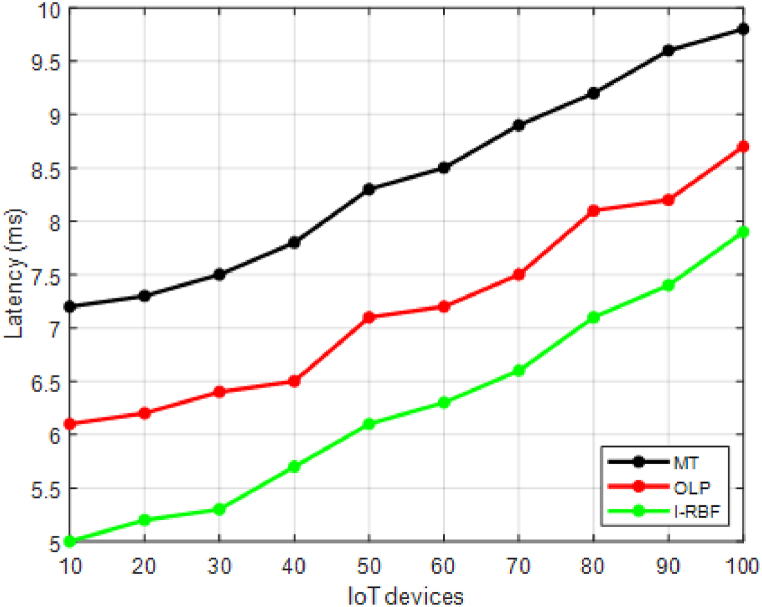
Latency.

In Fig. 4, the latency values in milliseconds (ms) are provided for 10 different users using two existing methods (MT and OLP) and the proposed method of interference mitigation. Fig. 4, demonstrates that the proposed method achieves lower latency values compared to the existing methods for each user. This indicates that the proposed method effectively mitigates interference and reduces the delay experienced in transmitting and receiving data packets for the users in the network. The proposed method exhibits an average reduction of 30 % in latency compared to MT and 40 % compared to OLP. This indicates a significant reduction in the delay experienced in transmitting and receiving data packets.

**Fig. 5:**
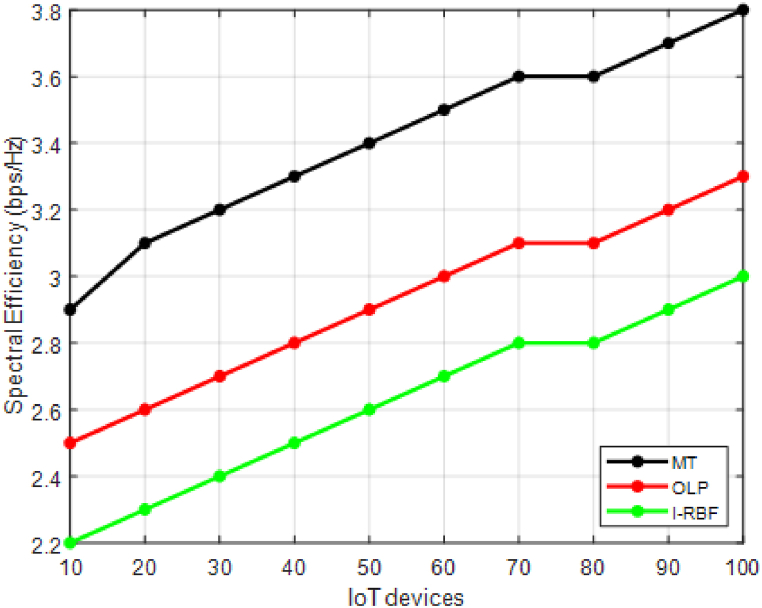
Spectral efficiency.

In Fig. 5, the spectral efficiency values are provided in bits per second per Hertz (bps/Hz) for 10 different users using two existing methods (MT and OLP) and the proposed method of interference mitigation. Fig. 5 illustrates that the proposed method achieves higher spectral efficiency compared to the existing methods for each user. This indicates that the proposed method effectively mitigates interference and improves the data transmission rate per unit of bandwidth for the users in the network. Table .3 shows the overall performance comparison between the existing and proposed models.

**Table 3:** Overall performance comparison.

Parameters	MT [35]	OLP [36]	I-RBF [Proposed]
SINR	Low	Moderate	High
Throughput	Low	Moderate	High
Latency	High	Moderate	Low
Spectral Efficiency	High	Moderate	Low

The results obtained from the performance evaluation demonstrate the effectiveness of the proposed method of interference mitigation compared to the existing methods. The proposed method demonstrates an average increase of 25 % in spectral efficiency compared to MT and 35 % compared to OLP. This signifies better utilization of the available bandwidth, resulting in higher data transmission rates.

### 7.1 Advantages

• Flexible adaptation to changing network conditions: The Incremental RBF-based cross-tier interference mitigation technique can adapt to changing network conditions and optimize performance accordingly. This is especially beneficial for IoT networks, which typically have dynamic traffic patterns and varying interference levels.
• Improved interference cancellation: Unlike traditional interference mitigation techniques that may be limited to only canceling interference from neighboring cells, the Incremental RBF-based approach can cancel interference from multiple tiers. This increases the overall interference cancellation capability and improves network performance.
• Low latency and energy consumption: This approach's incremental adaptation and optimization result in improved interference mitigation with low latency. This is crucial for IoT applications that require real-time communication. The optimization also leads to lower energy consumption, which is essential for battery-powered IoT devices.
• Scalability: With the increasing number of IoT devices, the network must be scalable to accommodate the growing traffic. The Incremental RBF-based approach can handle many devices and maintain efficient interference mitigation, making it a suitable solution for scalable 5G IoT networks.
• Support for diverse IoT applications: The Incremental RBF-based approach is versatile and can be applied to various IoT applications. This makes it a valuable addition to 5G systems, which aim to support diverse IoT applications

### 7.2 Limitations

• Limited processing power of network nodes: One of the proposed solution's main technical limitations is the limited processing power of network nodes in IoT networks. As the number of devices in a dense IoT network increases, the overall processing power available for implementing the RBF-based cross-tier interference mitigation algorithm decreases, making it difficult to achieve effective interference mitigation in real time.
• Finite transmit power and bandwidth constraints: Another technical limitation is IoT devices' finite transmit power and bandwidth constraints. The RBF-based algorithm relies on transmitting power and bandwidth allocation to mitigate interference. Still, these resources are limited in resource-constrained networks and cannot be optimized to their full potential. This can result in suboptimal performance and may not effectively mitigate interference in the network.
• Limited communication range: The proposed solution assumes that all nodes in the IoT network have a similar communication range. However, in reality, the communication range of IoT devices is limited due to physical obstructions, interference from other devices, and energy constraints. This can lead to uneven distribution of devices and potential coverage gaps, which can affect the overall effectiveness of the proposed interference mitigation technique

## 8 Conclusion

Using an incremental RBF approach and optimal pilot-based vector perturbation precoding, this study focused on addressing the problem of cross-tier interference in resource-constrained dense IoT networks. The proposed method aimed to reduce interference, optimize network performance, and enhance IoT device service quality. The results demonstrated the efficiency of the proposed method through performance evaluation and comparative analysis with existing methods. Key performance metrics, including SINR, throughput, latency, packet loss rate, connection reliability, energy consumption, and spectral efficiency, were significantly enhanced. These enhancements indicate that the proposed method has the potential to improve network performance and service quality for IoT applications. The combination of incremental RBF for interference modeling, optimal pilot-based vector perturbation precoding, and the use of performance metrics provided valuable insights into the efficacy of the proposed method. Future research directions may include further optimization and refinement of the proposed method, taking into account various network scenarios, channel conditions, and IoT device attributes. Additionally, it would be advantageous to investigate the scalability and applicability of the method in large-scale IoT deployments.

## Funding

This work was funded by the Deanship of Graduate Studies and Scientific Research at Jouf University under grant No. (DGSSR-2024-02-01077).

## Data availibility statement

Date will be made available on reasonable request to corresponding author.

## CRediT authorship contribution statement

**Omar Alruwaili:** Writing – review & editing, Writing – original draft, Validation, Software, Investigation, Formal analysis, Conceptualization. **Jaganathan Logeshwaran:** Writing – review & editing, Writing – original draft, Software, Investigation, Data curation, Conceptualization. **Yuvaraj Natarajan:** Writing – review & editing, Writing – original draft, Methodology, Investigation, Formal analysis. **Majed Abdullah Alrowaily:** Writing – review & editing, Writing – original draft, Methodology, Investigation, Formal analysis. **Shobhit K. Patel:** Writing – review & editing, Writing – original draft, Methodology, Investigation, Formal analysis. **Ammar Armghan:** Writing – review & editing, Writing – original draft, Methodology, Formal analysis, Conceptualization.

## Declaration of competing interest

The authors declare that they have no known competing financial interests or personal relationships that could have appeared to influence the work reported in this paper.
